# *MYT1L* mutations cause intellectual disability and variable obesity by dysregulating gene expression and development of the neuroendocrine hypothalamus

**DOI:** 10.1371/journal.pgen.1006957

**Published:** 2017-08-31

**Authors:** Patricia Blanchet, Martina Bebin, Shaam Bruet, Gregory M. Cooper, Michelle L. Thompson, Benedicte Duban-Bedu, Benedicte Gerard, Amelie Piton, Sylvie Suckno, Charu Deshpande, Virginia Clowes, Julie Vogt, Peter Turnpenny, Michael P. Williamson, Yves Alembik, Clinical Sequencing Exploratory Research Study Consortium, Deciphering Developmental Disorders Consortium, Eric Glasgow, Alisdair McNeill

**Affiliations:** 1 Département de Génétique Médicale, Hôpital Arnaud de Villeneuve, Montpellier Cedex 5, France; 2 Department of Neurology, University of Alabama at Birmingham, Birmingham, AL, United States of America; 3 Service de génétique médicale, Hôpitaux Universitaires de Clermont-Ferrand, Clermont-Ferrand, France; 4 Human Genetics and Genomics, HudsonAlpha Institute for Biotechnology, Huntsville, Alabama, United States of America; 5 Centre de génétique chromosomique, 51 Boulevard de Belfort, Lille, France; 6 Laboratoire de Diagnostic Génétique, Hôpitaux Universitaires de Strasbourg, Strasbourg, France; 7 Laboratoire de Diagnostic Génétique, Hôpitaux Universitaires de Strasbourg, 67000 Strasbourg, France; 8 Service de neuropédiatrie, Hôpital Saint Vincent de Paul, Lille, France; 9 South East Thames Regional Genetics Service, Guy's Hospital, London, United Kingdom; 10 North West Thames Regional Genetics Centre, Northwick Park Hospital, Harrow, United Kingdom; 11 Clinical Genetics Unit, Birmingham Women’s Hospital, Birmingham, United Kingdom; 12 Peninsula Clinical Genetics, Royal Devon & Exeter Hospital (Heavitree), Exeter, United Kingdom; 13 Department of Molecular Biology and Biotechnology, The University of Sheffield, Sheffield, United Kingdom; 14 Department of Medical Genetics, CHU Hautepierre, Strasbourg, France; 15 Wellcome Trust Sanger Institute, Wellcome Trust Genome Campus, Hinxton, United Kingdom; 16 Department of Medicine, Georgetown University, Washington, DC, United States of America; 17 Sheffield Institute for Translational Neuroscience, The University of Sheffield, Sheffield, United Kingdom; 18 Sheffield Clinical Genetics Service, Sheffield Children’s Hospital NHS Foundation Trust, Sheffield, United Kingdom; Murdoch Childrens Research Institute, AUSTRALIA

## Abstract

Deletions at chromosome 2p25.3 are associated with a syndrome consisting of intellectual disability and obesity. The smallest region of overlap for deletions at 2p25.3 contains *PXDN* and *MYT1L*. *MYT1L* is expressed only within the brain in humans. We hypothesized that single nucleotide variants (SNVs) in *MYT1L* would cause a phenotype resembling deletion at 2p25.3. To examine this we sought *MYT1L* SNVs in exome sequencing data from 4, 296 parent-child trios. Further variants were identified through a genematcher-facilitated collaboration. We report 9 patients with *MYT1L* SNVs (4 loss of function and 5 missense). The phenotype of SNV carriers overlapped with that of 2p25.3 deletion carriers. To identify the transcriptomic consequences of *MYT1L* loss of function we used CRISPR-Cas9 to create a knockout cell line. Gene Ontology analysis in knockout cells demonstrated altered expression of genes that regulate gene expression and that are localized to the nucleus. These differentially expressed genes were enriched for OMIM disease ontology terms “mental retardation”. To study the developmental effects of *MYT1L* loss of function we created a zebrafish knockdown using morpholinos. Knockdown zebrafish manifested loss of oxytocin expression in the preoptic neuroendocrine area. This study demonstrates that *MYT1L* variants are associated with syndromic obesity in humans. The mechanism is related to dysregulated expression of neurodevelopmental genes and altered development of the neuroendocrine hypothalamus.

## Author summary

Intellectual disability is defined by having an intelligence quotient of less than 70 points, and it affects about 2–3 people in every 100. Obesity is defined as having a body mass index of over 30 in adults or over the 95^th^ centile in children. Both of these conditions are major public health concerns in Western countries. Genetic studies have shown that small missing pieces of chromosome (deletions, which remove many genes) and changes to the lettering of genes (which stop the gene from working, mutations) can cause intellectual disability or obesity. Here we identified 9 children with intellectual disability and obesity who have mutations in a gene called *MYT1L*. This gene is thought to give an important instruction for brain development. To find out what the effect of loss of *MYT1L* is on brain development we reduced the levels of *MYT1L* (using a special chemical) in an experimental zebrafish. This showed that loss of *MYT1L* in zebrafish causes a problem with the development of the hypothalamus, which may explain how *MYT1L* mutations cause obesity in humans. In the zebrafish there was also reduction of a brain hormone called oxytocin which is involved in thought processes, which may explain why *MYT1L* mutations cause intellectual disability. We have identified a new genetic condition caused by *MYT1L* mutations, further study of this gene will help us understand, and treat, intellectual disability and obesity.

## Introduction

Intellectual disability (ID) is defined by having a full-scale intelligence quota (IQ) of under 70, causing difficulties with day to day functioning [1]. ID affects 2–3% of people and is a significant public health concern as it is associated with substantial morbidity and mortality [1]. Obesity is defined as a body mass index (BMI) of over 30 in adults or greater than the 95^th^ centile in children (CDC definition) [2]. Obesity affects around 30% of adults in the United States of America and 10–20% of Europeans [2]. Obesity is associated with cardiovascular disease and certain cancers [2].

Copy number variants (CNVs) and single nucleotide variants (SNVs) are a well-recognized cause of ID [3]. 10–30% of individuals with ID will have a pathogenic CNV [3]. Exome sequencing can detect pathogenic SNVs in around 30% of people with ID without a CNV [4]. Pathogenic CNVs and SNVs are also found in obesity, usually in association with a syndromic presentation [5]. For example, both CNVs and SNVs of *SIM1* are associated with obesity in humans [6, 7]. In *SIM1* deletion heterozygous mice there is impaired development of the paraventricular nucleus of the hypothalamus, with reduced melanocortin-4 receptor and oxytocin expression, in association with hyperphagic obesity [8, 9].

Deletions at 2p25.3 are associated with a syndrome consisting of ID and obesity [10,11,12,13,14]. The smallest region of overlap contains the *PXDN* and myelin transcription factor-1 like (*MYT1L*) genes [10]. Bi-allelic SNVs in *PXDN* are associated with congenital cataracts [15]. No CNVs containing only *PXDN* have been reported in DECIPHER in association with ID. Thus *PXDN* is not a strong candidate gene for the phenotype associated with 2p25.3 deletions. SNVs in *MYT1L* have been reported in 2 children with ID [10]. *MYT1L* is a member of the myelin transcription factor family, which is defined by the presence of a unique cystein-cystein-histidine-cystein zinc finger domain [16]. *MYT1L* is a pro-neuronal transcription factor, and, in combination with other transcription factors can re-program fibroblasts into neurons [17]. In vitro studies indicate that *MYT1L* functions as a transcriptional repressor [16]. The role of *MYT1L* in brain development is not well understood. However, myelin transcription factor-1 (*MYT1*) has been shown to repress transcription in neuronal progenitor cells, hence blocking Notch signaling and promoting neuronal differentiation [18]. Based upon its biological function *MYT1L* is a strong candidate gene for ID.

Within certain deletion regions, there are single genes in which SNVs recapitulate the deletion phenotype. For example, we recently demonstrated that 2p25.2 deletions and SNVs in *SOX11* present with Coffin-Siris syndrome [19]. Here we utilized exome-sequencing data from 4,296 parent-child trios in the Deciphering Developmental Disorders (DDD) study to demonstrate that SNVs in *MYT1L* are associated with a phenotype resembling that of 2p25.3 deletions with ID and obesity [4]. Through gene expression profiling of an *MYT1L* null cell line we show that *MYT1L* regulates a network of transcription factors involved in neurodevelopmental disorders. Knockdown of *MYT1L* orthologues in zebrafish resulted in altered hypothalamic oxytocin expression, providing a potential mechanism for the obesity phenotype in humans.

## Results

### Exome sequencing identifies *MYT1L* variants in individuals with intellectual disability

**Fig 1:**
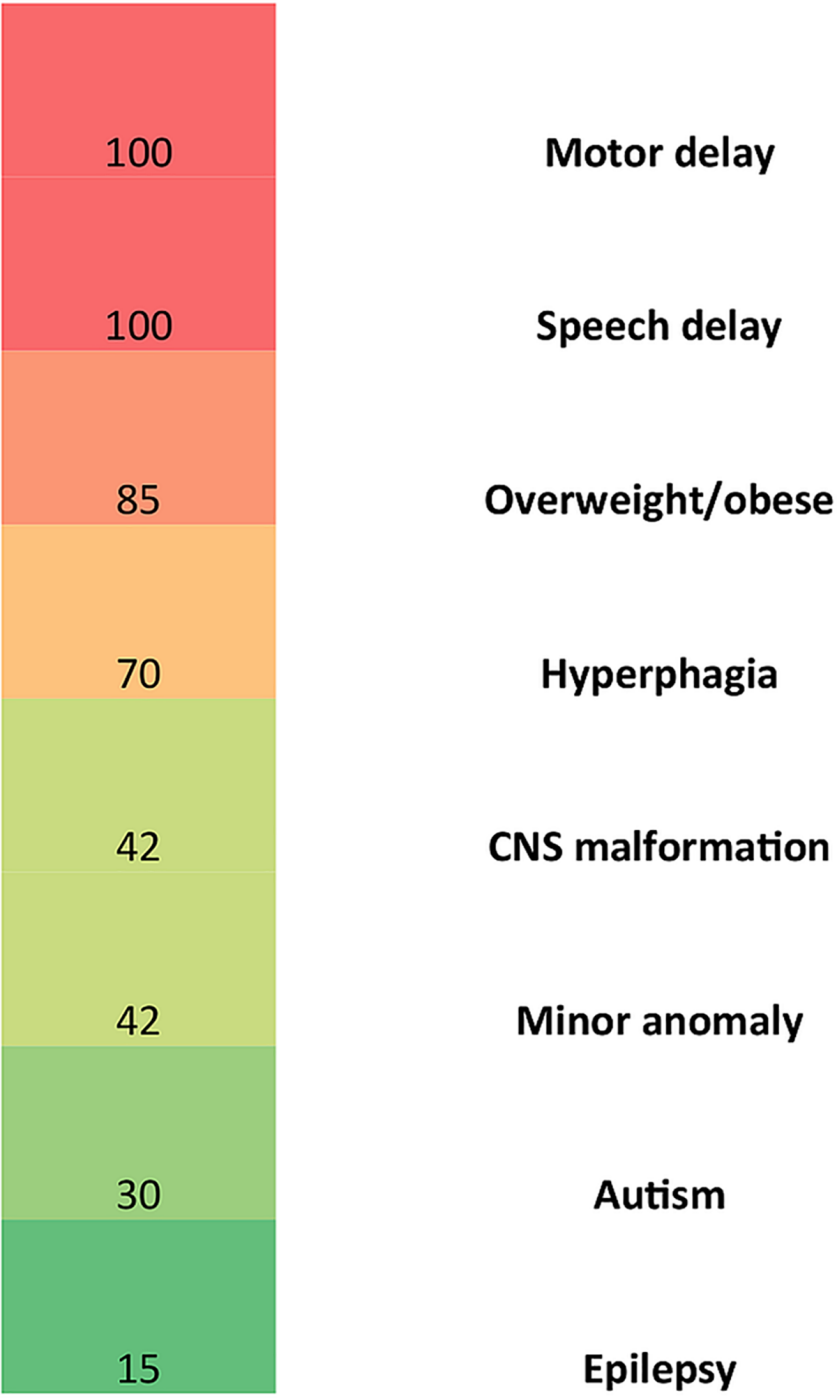
Clinical characterization of *MYT1L* variant carriers. The heatmap depicts the frequency of various clinical features in *MYT1L* variant carriers.

We identified 4 individuals with heterozygous *de novo MYT1L* variants through trio exome sequencing performed as part of the Deciphering Developmental Disorders study and an additional 5 individuals with heterozygous *de novo MYT1L* variants through a genematcher facilitated collaboration (https://genematcher.org/)[20]. Table 1 and Fig 1 summarize the clinical and genetic findings.

**Table 1:** Summary of demographic features, developmental milestones, medical complications and exome sequencing of cohort.

	Patient 1	Patient 2	Patient 3	Patient 4
Demographics	F, 10 years	M, 9 years	M, 10 years	F, 28 years
Whole exome result	g.1926242CA>C p.Leu381fs	g.1915795C>T p.Arg569Gln	g.1915823G>A p.His560Tyr	g.1915791C>T Splice donor variant
OFC (cm)	55.6 cm (75^th^)	54.5 cm (75^th^)	54cm (42^nd^)	55cm (40^th^)
Ht (cm)	121.8 (0.4^th^)	146.7 (98^th^)	136.6 (50^th^)	N/A
Weight (kg)	46.5 (98^th^)	43.4 (99^th^)	34.9 (75^th^)	N/A
BMI	>99^th^ centile	96^th^ centile	85^th^ centile	Dress size 22
Development	Sitting 12 months Single steps with support 6 years Not walking independently Single words 5 years	Single words 15 months	Sitting 18 months Walked independently 24 months Single words 5 years	Sitting 18 months Single words 4 years Non-ambulant (neural tube defect)
Appetite	Hyperphagia	Hyperphagia	Hyperphagia	N/A
Pregnancy	Born at 31 weeks, long period in SCBU, NGT fed, floppy.	Normal	Normal	Normal
Tests	CGH normal MRI–white matter thinning SNRPN normal EMG/NCS normal.	CGH normal MRI–cerebral atrophy	CGH normal MRI brain Normal	CGH normal
Other	Bilateral congenital talipes Bilateral squint Autism	Mild gait ataxia	anioshypermetropic astygmatism	Self-mutilation Neural tube defect Hydrocephalus Autism
	Patient 5	Patient 6	Patient 7	Patient 8
Demographics	F, 17 years	F, 15 years	F, 5 years	F, 3 years
Whole exome result	g.1921036A>G p.Leu520Pro	g.1895856_1895865del p.Thr741fs	g.1890345G>A p.Arg839Ter	g.1921025G>T p.His524Asn
OFC (cm)	N/A	52.5 cm (2^nd^)	51.5 cm (25^th^)	51 cm (75^th^)
Ht (cm)	160 cm (25^th^)	163 cm (50^th^)	119cm (98^th^)	99cm (91^st^)
Weight (kg)	78.8 kg (98^th^)	59 kg (62^nd^)	30kg (99.6^th^)	16kg (98^th^)
BMI	>99^th^ centile	78^th^ centile	>99^th^ centile	75^th^ centile
Development	Delayed speech Motor delay	Delayed speech Walked 22 months Special school	Sat 9 months Walked 19 months Single words 2 years Poor fine motor skills 5 years Extra help at school	Sat 9 months Walked 23 months Running 36 months No speech
Appetite	Hyperphagia	normal	Hyperphagia	normal
Pregnancy	Normal	Normal	Normal	normal
Tests	CGH normal MRI brain Normal	CGH normal	Paternal 20p12 dup	MRI: thin corpus callosum
Other	Complex partial seizures from age 11 years Autism		Hyperactive	Hypotonia Absence seizures Autism
	Patient 9	Patient 10		
Demographics	M, 13 years	M, 27 months		
Whole exome result	c.1579G>A p.Gly527Arg	2.3 Mb 2p25.3 deletion		
OFC (cm)	53cm (25^th^)	50cm (50^th^)		
Ht (cm)	149cm (40^th^)	80.5 cm (0.4^th^)		
Weight (kg)	44kg (40^th^)	16kg (99.6^th^)		
BMI	77^th^	>99^th^ centile		
Development	First sat 12 months Walked 22 months Single words 29 months	Not walking age 2 No speech		
Appetite	Normal	Hyperphagia		
Pregnancy	Normal	Normal		
Tests	CGH normal MRI brain normal	N/A		
Other	Scoliosis ADHD Poor sleep			

Abbreviations used: ADHD (Attention deficit hyperactivity disorder), OFC (orbitofrontal circumference), Ht (height) BMI (body mass index), CGH (comparative genomic hybridization), MRI (magnetic resonance imaging), EMG (electromyography), NCS (nerve conduction studies), SCBU (special care baby unit), NGT (nasogastric tube).

Patient 1 (DECIPHER ID 268494) is a 10 year old girl with intellectual disability and autism. She was born at 31 weeks of gestation with bilateral talipes equinovarus and camptodactyly of the ring and middle fingers. Up until 5 years of age she had troublesome gastro-esophageal reflux. She first sat at 12 months old. From age 6 years she could take single steps with support. She requires a wheelchair and has never walked independently. She first said single words at 5–6 years old and at 10 years old speaks in simple sentences. She has dysarthria. She has had surgery for bilateral strabismus. Her parents reported hyperphagia and her BMI was greater than the 99^th^ centile. Comparative genomic hybridization (CGH) and *SNRPN* 15q methylation (for Prader-Willi syndrome) were normal. Exome sequencing demonstrated a frameshift variant in *MYT1L* (g.1926242CA>C, p.Leu381fs).

Patient 2 (DECIPHER 279017) is a 9 year old boy with intellectual disability who attends a special needs school. Pregnancy and birth were unremarkable. He first spoke single words at 15 months. On examination he was noted to have posterior plagiocephaly, 5^th^ finger clinodactyly and an ataxic gait. He was not dysmorphic. His parents described him as having hyperphagia and his BMI was on the 96^th^ centile. At age 9 he wears clothes for an 11–12 year old. CGH was normal and brain magnetic resonance imaging (MRI) demonstrated cerebral atrophy. Exome sequencing demonstrated a missense variant in *MYT1L* (g.1915795C>T, p.Arg569Gln).

Patient 3 (DECIPHER ID 279061) is a 10 year old boy with intellectual disability, Attention Deficit Hyperactivity Disorder (ADHD) and verbal dyspraxia. He sat at 18 months and walked first at 2.5 years. He first said single words at 4–5 years old. At 10 years old he uses 2 word phrases, but mainly communicates with sign language. He wears glasses for anioshypermetropiic astigmatism. He was described as having hyperphagia and his BMI was on the 85^th^ centile. CGH, fragile X, PWS testing and brain MRI were normal. Exome sequencing demonstrated an *MYT1L* missense variant (g.1915823G>A, p.His560Tyr)

Patient 4 (DECIPER ID 276823) is a 28 year old woman with severe intellectual disability, autism, self-injurious behavior and ADHD. She was born at 42 weeks of gestation with spina bifida. She developed hydrocephalus in the first week of life. She has a ventricular shunt and has required several procedures for shunt blockage. She sat at 18 months. She smiled at 3 weeks. She said single words at 4–5 years. She requires a wheelchair because of spastic paraparesis due to spina bifida. She is not dysmorphic. Her behavior is reported to be challenging, including episodes of biting and pinching, triggered by excessive stimuli such as noise or crowding. She has limited verbal communication, and uses communication aids. She can comprehend short (3 key phrases) sentences. Due to her level of disability she does not request or seek food. However, when given food at mealtimes, she was noted to have a tendency to fill her mouth excessively with food while eating. It was not possible to obtain weight or height. However, she wears UK dress size 22 clothes (equivalent to dress size 20 in United States of America, dress size 50 in Europe and dress size 24 in Australia). CGH was normal. Exome sequencing demonstrated an *MYT1L* splice donor variant (g.1915791C>T).

Patient 5 is a 17 year old woman with intellectual disability and autism. She was born at 41 weeks gestation with no birth complications. Gross motor, fine motor and speech delay was noted at 2 and 4 years old. She is noted to have dyslexia. She was not dysmorphic. She had complex partial seizures from the age of 11 years old. She was reported to have hyperphagia and BMI was greater than the 99^th^ centile. CGH and brain MRI were normal. Whole genome sequencing demonstrated an *MYT1L* missense variant (g.1921036A>G, p.Leu520Pro).

Patient 6 is a 15 year old girl with intellectual disability who attends a special needs school. She first walked at 22 months old and had delayed speech. She was not dysmorphic. She did not have hyperphagia and her BMI was on the 74^th^ centile. CGH was normal. Exome sequencing demonstrated an *MYT1L* frameshift variant (g.1895856_1895865del, p.Thr741fs).

Patient 7 is a 5 year old girl with intellectual disability who required additional help at school. She was born at term with no birth complications. She first sat at 9 months. She walked first at 19 months. She required physiotherapy. She had speech delay, speaking single words after 2 years of age. At 5 years old she had ongoing speech and language delay and was reported to be clumsy. She was not dysmorphic. Eye examination demonstrated hyperopia and strabismus. She had hyperphagia and her BMI was greater than the 99^th^ centile. CGH demonstrated a 20p12 duplication, inherited from her phenotypically normal father. Exome sequencing demonstrated an *MYT1L* nonsense variant (g.1890345G>A, p.Arg839Ter).

Patient 8 is a 3 year old girl with global developmental delay and autistic behaviour. She was born at term with no pregnancy or birth complications. She had global hypotonia during the first few months of life. She had global developmental delay. She first sat at 9 months of age, walked independently at 2 years of age. At age 3 she had not developed speech, but used sign language. At age 3 she was beginning to learn to run. She could draw a line and a circle. She had absence seizures. Her BMI was on the 75^th^ centile and hyperphagia was not present. MRI brain demonstrated thinning of the corpus callosum. Exome sequencing demonstrated an *MYT1L* missense variant (g.1921025G>T, p.His524Asn).

Patient 9 is a 13 year old boy with global developmental delay and ADHD. Pregnancy and birth were unremarkable. Hypotonia and poor sleep were noted in the neonatal period. He first sat at 12 months of age and walked at 22 months. He spoke single words at 29 months of age. At 5 years old he knew 20 words, but had pronunciation difficulties. At 7 years old he knew 50 words and was using 2-word sentences. At the age of 13 he was unable to read, write or count. He attends a special educational needs school. His BMI was on the 68^th^ centile. A next generation sequencing gene panel test demonstrated an *MYT1L* missense variant (c.1579G>A, p.Gly527Arg).

### The *MYT1L* gene is constrained for missense and protein truncating variants

**Fig 2:**
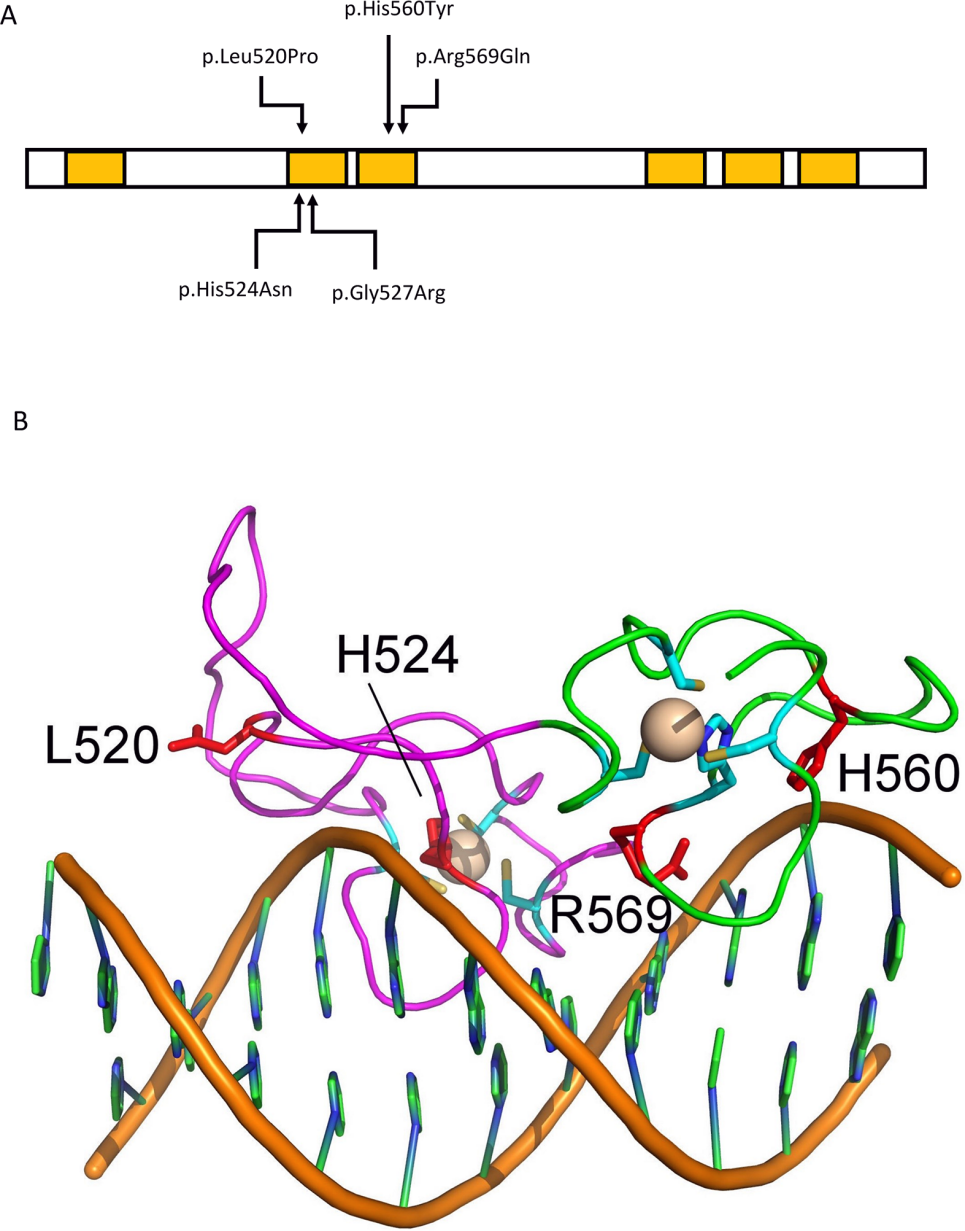
Structural effects of *MYT1L* missense variants. (A)Schematic diagram of MYT1L protein, the yellow boxes indicate zinc finger domains. The missense variants are indicated by arrows. (B)Model of the 2^nd^ and 3^rd^ zinc fingers of MYT1L bound to DNA. This is based upon the structure of the 4^th^ and 5^th^ zinc fingers of MYT1 (protein data bank file 2mf8), which have high sequence similarity to the 2^nd^ and 3^rd^ zinc fingers of MYT1L. The second zinc finger is in magenta, and the third finger in green. The beige spheres represent the zinc ions, with the CCHC zinc ligands shown in cyan. Replacement of L520 by proline is expected to disrupt the structure of the protein by preventing the formation of a tight turn. H524 and G527 are a zinc ligands, so replacement will also disrupt the structure. H560 and R569 are conserved residues directly involved in DNA binding. Image created using Pymol.

Protein truncating variants (PTV) in developmentally crucial genes should occur less frequently than predicted in individuals without developmental disorders. The expected frequency of PTV in human genes has been reported in the ExAC database based upon parameters such as mutation rates for given nucleotide bases [21]. We identified only a single loss of function variant in *MYT1L* in the ExAC database (accessed March 2017) [21]. This is compared to an expected number of 33 loss of function variants, giving a probability of loss of function intolerance score of 1.0 (a probability of loss of function intolerance score>0.9 indicates intolerance to loss of function). In addition, 205 missense variants were reported, compared to an expected 402.5 missense variants (Z score = 4.81, indicating constraint on variation). The Provean (median -5.2 [interquartile range -6.0 - -3.85] vs -1.1 [interquartile range -2.59 - -0.43], Mann-Whitney U-test, u = 344, z = -2.96, p = 0.012) and SIFT (median 0.001 [interquartile range 0.005–0.28] vs 0.068 [interquartile range 0.0003–0.003], Mann-Whitney U-test, u = 569, z = -2.61, p = 0.025) scores for the missense variants in our patients were significantly higher than the scores for ExAC missense variants (Fig 2A). This indicates substantial constraint on both PTV and missense variants. This supports a pathogenic role for PTV and missense variants in the reported phenotype. In addition, an *in silico* model of the structural effects of the *MYT1L* missense variants indicated that they were likely to interfere with DNA binding (Fig 2B).

### The phenotype of *MYT1L* variant carriers resembles that of 2p25.3 microdeletion patients

To examine the hypothesis that haploinsufficiency for *MYT1L* drives the 2p25.3 deletion syndrome, we compared the phenotype of 2p25.3 deletion carriers with those of *MYT1L* single nucleotide variant (SNV) carriers. The phenotypes associated with deletion of 2p25.3 were defined by a literature review [10, 11, 12, 13, and 14] and we report an unpublished case (patient 10 in Table 1). The smallest region of overlap contains *PXDN* and *MYT1L*. No *PXDN* SNVs were identified in the DDD exome dataset. Using Fischer’s exact test there was no significant difference between the proportions of deletion or SNV patients with the following phenotypes: intellectual disability, gross motor delay, speech delay, autism, overweight/obese or hyperphagia. This supports our hypothesis that *MYT1L* haploinsufficiency is central to the 2p25.3 deletion phenotype.

### Expression of *MYT1L* occurs in brain regions relevant to the human disease phenotype

Given the phenotype of intellectual disability and predisposition to overweight/obesity we reasoned that *MYT1L* should be expressed in relevant neuroanatomical structures. We first confirmed that *MYT1L* expression is confined to the brain and pituitary in humans using the GTEx Portal (accessed March 2017) [22]. We then utilized the Allen Brain atlas to examine the spatial expression pattern of *MYT1L* in human brain [23].

**Fig 3:**
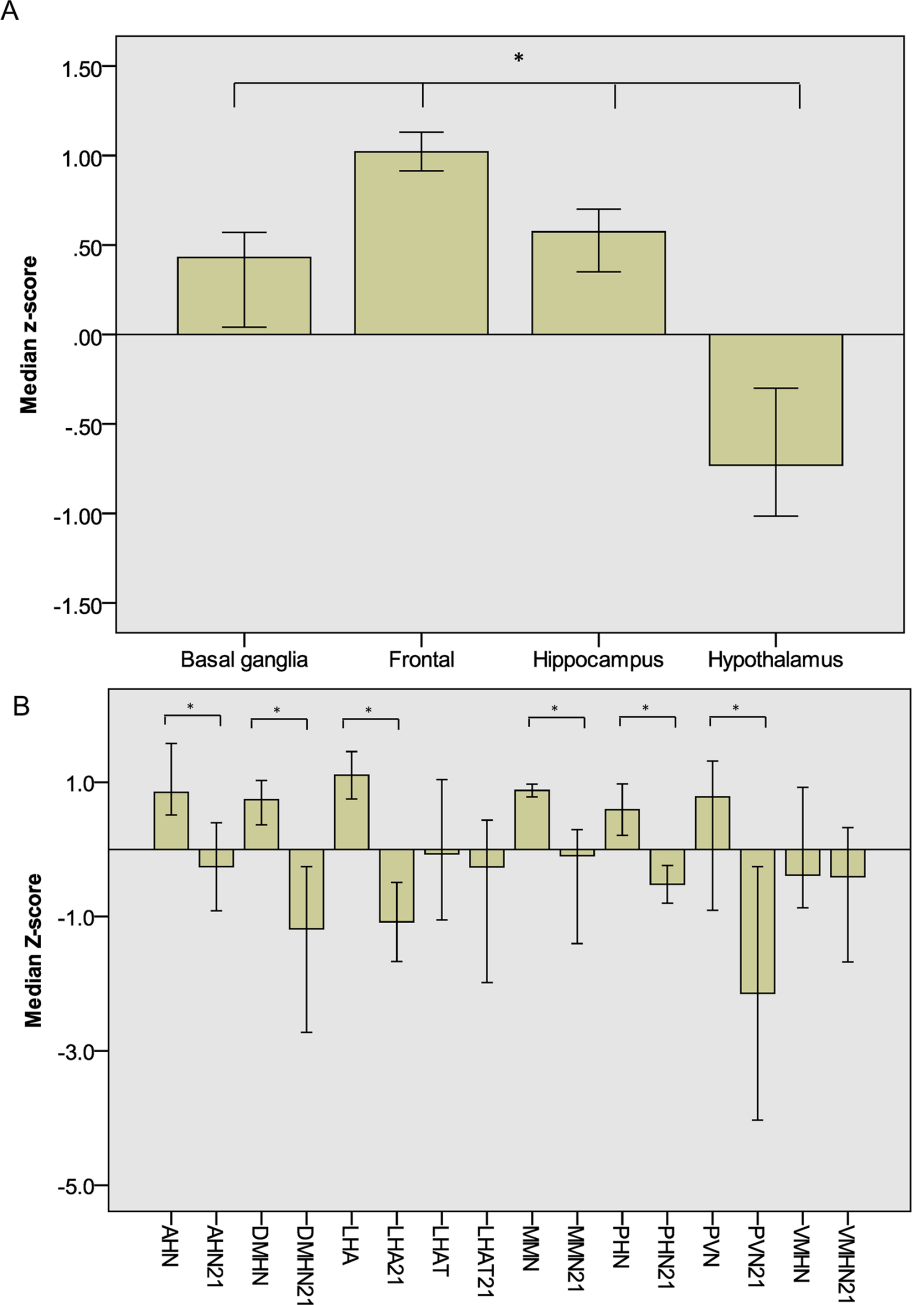
*MYT1L* expression in human brain. (A) *MYT1L* expression levels are significantly higher in the frontal cortex than hippocampus, basal ganglia or hypothalamus in adult brain (p<0.001). Data presented as median z-score +/- 95% confidence interval. (B) *MYT1L* expression levels in the developing hypothalamus are significantly higher at 15–16 post conception weeks than 21 post conception weeks (p<0.001). The labels on the x-axis with 21 indicate expression at 21 post conception weeks, the x-axis labels without 21 indicate expression at 15–16 weeks. Data presented as median z-score +/- 95% confidence interval AHN = anterior hypothalamic nucleus. DMHN = dorsomedial hypothalamic nucleus. LHA = lateral hypothalamic area. MMN = medial mammillary nucleus. PHN = posterior hypothalamic nucleus. PVN = paraventricular nucleus. VMHN = venteromedial hypothalamic nucleus.

In keeping with a role in cognition/intellectual disability, *MYT1L* is expressed at significantly higher levels in the adult cerebral cortex than in the hippocampus, basal ganglia and hypothalamus (Mann-Whitney U-test, P<0.001)(Fig 3A). We could not demonstrate significant expression of *MYT1L* in hypothalamic structures relevant to appetite and obesity in the adult brain.

We then hypothesized that *MYT1L* might be expressed in hypothalamic structures relevant to appetite and obesity during brain development. Data from the prenatal LMD microarray from the Brainspan atlas of the developing human brain demonstrated that *MYT1L* was expressed in multiple hypothalamic structures at 15–16 post conception weeks (pcw), with significant reduction in expression at 21 pcw (Mann-Whitney U-test, p<0.001)(Fig 3B). This suggests that *MYT1L* principally plays a role in hypothalamic development rather than postnatal hypothalamic function.

### Loss of *MYT1L* expression dysregulates expression of genes involved in neurodevelopmental disorders

**Fig 4:**
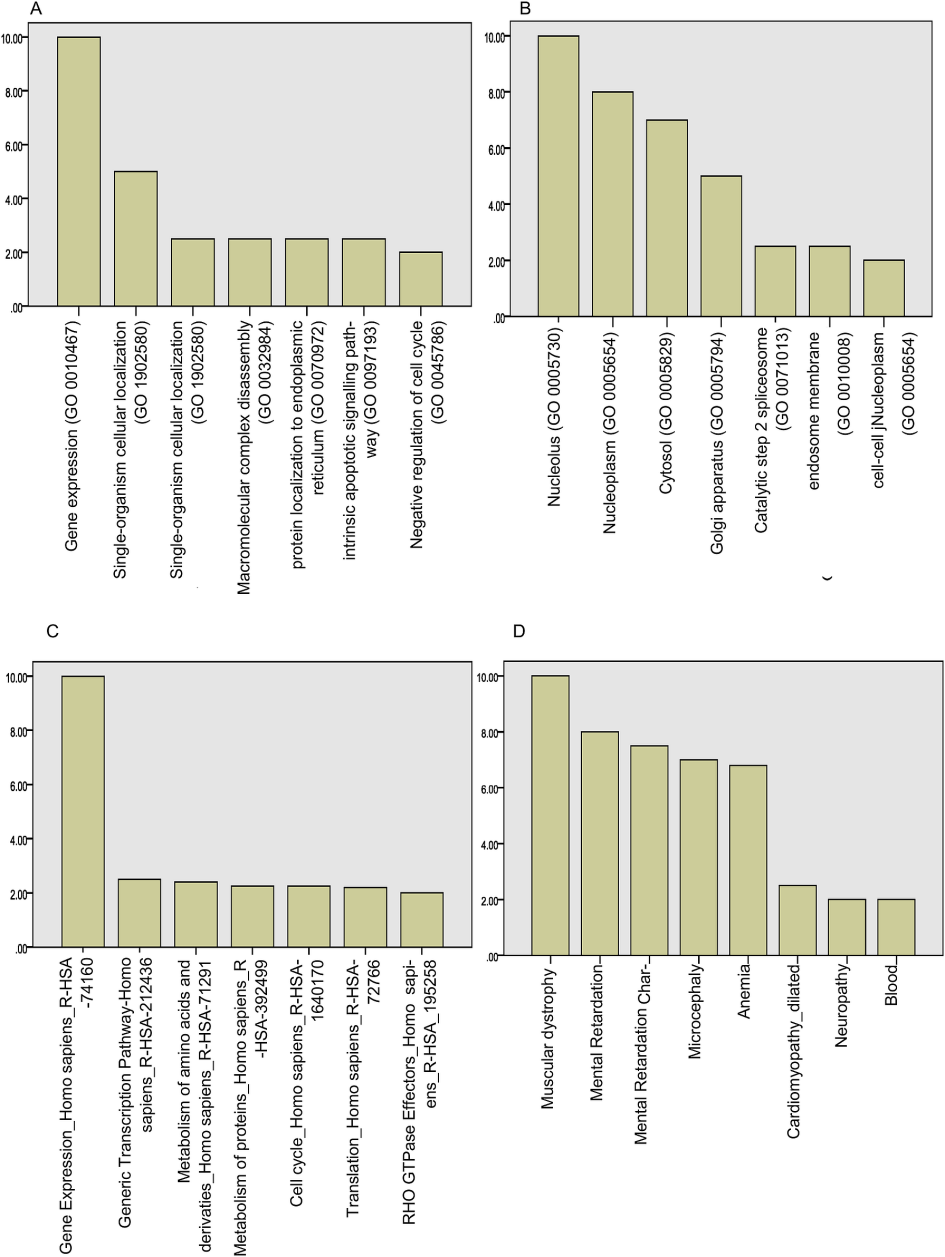
Gene expression profiling of *MYT1L* knockout cell line. (A)Enrichment analysis demonstrates enrichment for Gene Ontology Biological Process 2015 term gene expression (GO:0010467, adjusted p-value 0.00077, Z-score -2.34, combined score 16.77). (B)Enrichment analysis demonstrates enrichment for Gene Ontology Cellular Component 2015 terms nucleolus (GO: 0005730, adjusted p-value 0.0023, Z-score -2.21, combined score 13.43) and nucleoplasm (GO: 0005654, adjusted p-value 0.005853, Z-scre -2.08, combined score 11.4). (C)Enrichment analysis demonstrates enrichment for Reactome 2016 pathways Gene Expression_Homo Sapiens_R-HAS-74160 (adjusted p-value 2.2 x 10–7, Z-score -2.16, combined score 33) and Generic Transcription Pathway_Homo Sapiens_R-HAS-212436 (adjusted p-value 0.01586, Z-score -2.26, combined score 9.35). (D)Enrichment analysis demonstrates enrichment for OMIM disease term mental retardation (p-value 0.045, adjusted p-value 0.38, Z-score -1.32, combined score 1.27).

To study the transcriptional consequence of loss of *MYT1L* function we created a Human Embryonic Kidney (HEK) null cell line using CRISPR-Cas9. Sanger sequencing of genomic DNA confirmed creation of homozygous premature stop codons in *MYT1L*. Gene expression profiling using the Clariom S array identified 955 differentially expressed genes (2-fold expression change, false discovery rate 2%). Enrichment analysis (using Enrichr http://amp.pharm.mssm.edu/Enrichr/) [24]demonstrated that the differentially expressed gene set was enriched for the Gene Ontology Biological Process 2015 term gene expression (GO:0010467, adjusted p-value 0.00077, Z-score -2.34, combined score 16.77)(Fig 4A) and Gene Ontology Cellular Component 2015 terms nucleolus (GO: 0005730, adjusted p-value 0.0023, Z-score -2.21, combined score 13.43) and nucleoplasm (GO: 0005654, adjusted p-value 0.005853, Z-score -2.08, combined score 11.4) (Fig 4B). The gene set was also enriched for the Reactome 2016 pathways Gene Expression_Homo Sapiens_R-HAS-74160 (adjusted p-value 2.2 x 10–7, Z-score -2.16, combined score 33) and Generic Transcription Pathway_Homo Sapiens_R-HAS-212436 (adjusted p-value 0.01586, Z-score -2.26, combined score 9.35)(Fig 4C). The differentially expressed genes were also enriched for genes from the OMIM disease ontology term mental retardation (p-value 0.045, adjusted p-value 0.38, Z-score -1.32, combined score 1.27)(Fig 4D). This suggests that *MYT1L* regulates a network of genes that control transcription, and which have themselves been implicated in the etiology of neurodevelopmental disorders.

### The zebrafish *MYT1L* orthologs (*myt1la* and *myt1lb*) are expressed only in the central nervous system

**Fig 5:**
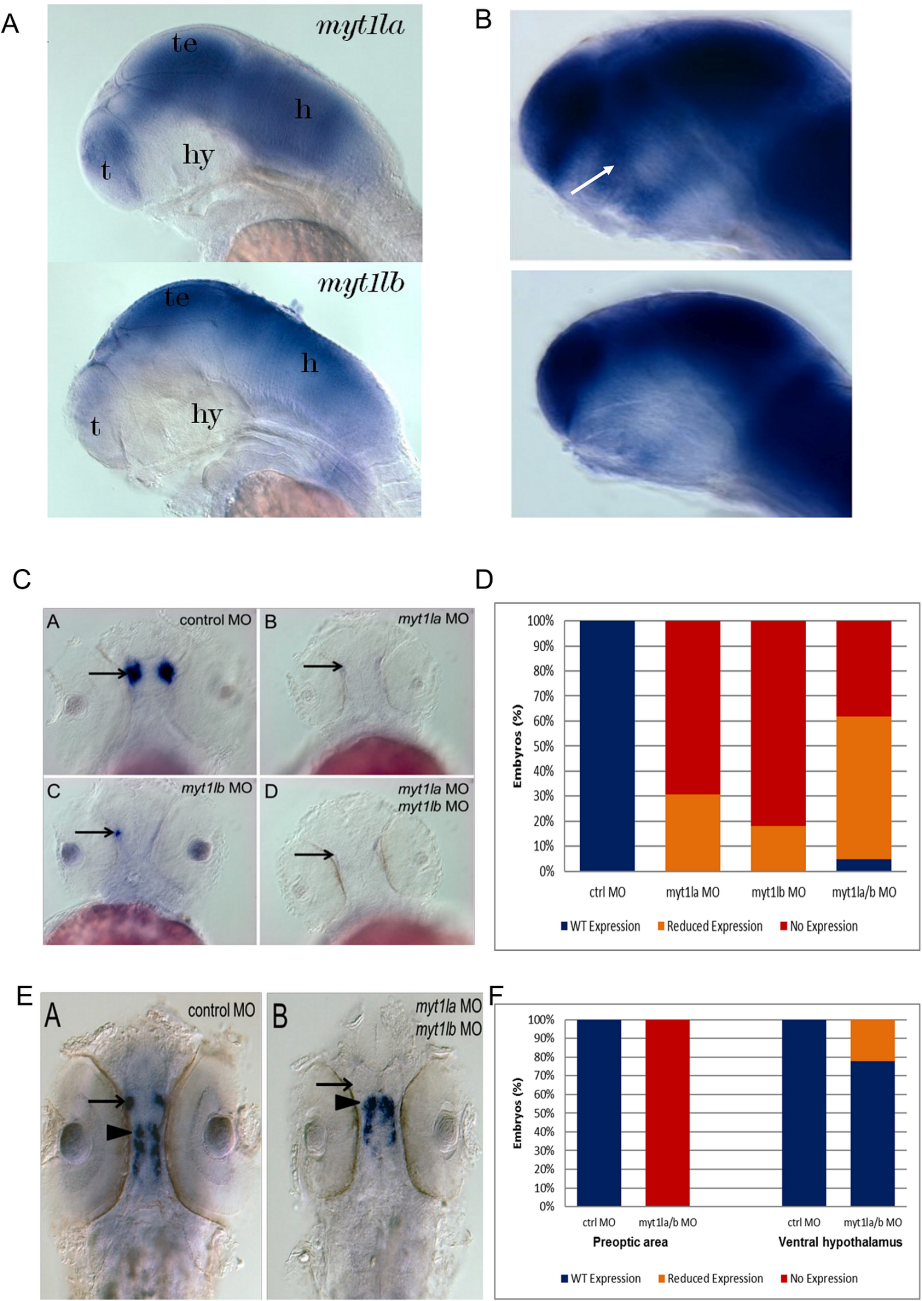
Hypothalamic peptide expression in zebrafish knockdown for *MYT1L* orthologues. (A)Whole mount in situ hybridization demonstrating that *MYT1L* orthologs *myt1la* and *myt1lb* are expressed throughout the zebrafish central nervous system. T = telencephalon, te = tectum, hy = hypothalamus, h = hindbrain. (B)Whole mount in situ hybridization demonstrating loss of *myt1la* expression in *arnt2* mutant zebrafish, top panel shows control fish and bottom panel *arnt2* mutant fish. The embryos are heavily over-stained to show the low-level expression of *myt1la* in the ventral diencephalon. The arrow indicates the region of the neuroendocrine preoptic area where oxytocin expressing neurons are located. (C)Whole mount in situ hybridization demonstrating that knockdown of *myt1la* and *myt1lb*, alone or in combination, results in loss of oxytocin expression in the neuroendocrine preoptic area. Arrows indicate neuroendocrine preoptic area. (D)Bar chart quantifying loss of oxytocin expression in neuroendocrine preoptic area. WISH for oxytocin was quantified as follows: ~30 cells = wild type expression, 5–15 cells = reduced, 1–4 = highly reduced, 0 = no expression (E)Whole mount in situ hybridization demonstrating that knockdown of *myt1la/b* results in loss of arginine vasopressin expression in the neuroendocrine preoptic region (indicated by arrow) but not the ventral hypothalamus (indicated by arrowhead). (F) Bar chart quantifying loss of arginine vasopression expression in *myt1la/b* morphants. WISH for arginine vasopressin was quantified as follows: ~30 cells = wild type expression, 5–15 cells = reduced, 1–4 = highly reduced, 0 = no expression.

Given the obesity phenotype in patients with *MYT1L* SNVs we hypothesized that loss of *MYT1L* function may interfere with development of the neuroendocrine hypothalamus. We sought to explore this by creating a zebrafish knockdown model. *MYT1L* has two orthologs in zebrafish: *myt1la* and *myt1lb*. By using whole mount in situ hybridization (WISH) we demonstrate that both orthologs are expressed diffusely within the zebrafish central nervous system, including the hypothalamus, and not within any extra-neuronal tissues (Fig 5A). This resembles the expression pattern in humans.

### Loss of sim1 function results in reduced myt1la expression in zebrafish

*ANRT2* encodes a dimerization partner required for *SIM1* function in hypothalamic development. To examine the role of SIM1-ARNT2 in regulating *myt1la/mytl1b* we performed WISH for *myt1la* in the previously described homozygous *arnt2*^*hi2639Tg*^ null zebrafish [25]. In the mutant zebrafish *myt1la* was undetectable in the hypothalamus (Fig 5B). This shows a role for SIM1-ARNT2 dimers in regulating *myt1la* expression in the hypothalamus. These experiments demonstrate that *MYT1L* lies downstream of *SIM1-ARNT2* in the leptin-melanocortin-SIM1 pathway, and, in turn, *MYT1L* regulates *OXT* expression in the hypothalamus.

### Knockdown of *myt1la/myt1lb* causes altered hypothalamic development in zebrafish

Antisense morpholinos (MO) targeting *myt1la or myt1lb*, alone or in combination, were injected into zebrafish embryos at the 1- or 2-cell stage. The amount of MO injected was kept constant. WISH using probes against oxytocin (*OXT*) or arginine vasopression (*AVP*) was performed. MO targeting *myt1la* or *myt1lb* resulted in an almost complete loss of *OXT* in the neuroendocrine pre-optic area (Fig 5C and 5D). MO targeting *myt1la* or *myt1lb* also resulted in an almost complete loss of *AVP* in the neuroendocrine pre-optic area, but not in the ventral hypothalamus (Fig 5E and 5F). This suggests that *myt1la* and *myt1lb* may play a specific role in regulating the development of the neuroendocrine hypothalamus.

## Discussion

Here we describe 9 individuals with *de novo* SNVs in *MYT1L*. These individuals shared several phenotypic features. All had global developmental delay or intellectual disability. Gross motor delay was present in all, and patient 1 had not walked independently by the age of 10 years old. Patient 4 also required a wheelchair, but this was related to spastic paraparesis secondary to spina bifida. No other individuals with CNVs or SNVs of *MYT1L* have been reported with a neural tube defect. The spina bifida in patient 4 is likely etiologically unrelated to the *MYT1L* SNV. Six of the *MYT1L* SNV carriers were overweight or obese based upon BMI centiles and patient 4 requires a dress size 22, implying obesity. Five of the patients had hyperphagia, but patient 4 did not have sufficient speech development or motor function to ask for or take food. Three had a diagnosis of an autism spectrum disorder. There was no shared, distinctive facial dysmorphology. Two *MYT1L* SNV carriers have been reported by De Rocker *et al*: patient 14 had developmental delay, autism and was described as obese (BMI not reported), patient 15 had developmental delay, autism and BMI >97^th^ centile [10]. An autistic male with developmental delay and febrile seizures in association with an *MYT1L* nonsense mutation was reported by Wang et al [26], however BMI was not described in this paper. There was no significant difference in the frequency of phenotypic features between 2p25.3 deletion and *MYT1L* SNV carriers. This suggests that haploinsufficiency for *MTY1L* drives the 2p25.3 deletion phenotype. Our report confirms that CNVs and SNVs of *MYT1L* are associated with a syndromic presentation consisting of developmental delay/ID, hyperphagia and obesity.

The clinical presentation of *MYT1L* CNV and SNV carriers overlaps with other mendelian causes of obesity. Prader-Willi syndrome (PWS) is a well-recognized cause of hyperphagic obesity [27]. The presence of characteristic dysmorphology and hypogenitalism may help differentiate PWS from *MYT1L* SNV/CNV carriers [27]. Deletions and SNVs of *SIM1* are associated with hyperphagic obesity [6, 7], with developmental delay associated with 6q14.1 deletions [7]. Bi-allelic SNVs of leptin (*LEP*) [28], leptin receptor (*LEPR*) [29], and pro-opiomelanocortin (*POMC*) [30] are also associated with hyperphagic obesity. *LEP* and *LEPR* can be distinguished from *MYT1L* due to the association of hypogonadotrophic hypogonadism with *LEP* and *LEPR* SNVs. Patients with *POMC* deficiency present with a range of endocrine problems not reported in association with *MYT1L* variants. There is also phenotypic overlap between certain microdeletion syndromes and *MYT1L*. Smith-Magenis syndrome (17p11.2 deletion) is associated with developmental delay and variable obesity [31]. Facial dysmorphology, sleep disturbance and self-injurious behavior reported in Smith-Magenis syndrome were not identified in our *MYT1L* cohort. 22q11.2 deletion carriers have increased rates of obesity [32], and the presence of cleft lip/palate, congenital heart disease or parathyroid disease can permit distinction from *MYT1L* variant carriers.

Our data supports the hypothesis that *MYT1L* SNVs cause loss of protein function and haploinsufficiency. Data from the ExAC indicated a loss of function intolerance score of one, which indicates that *MYT1L* is a haploinsufficient gene that will not tolerate heterozygous loss of function variants. Four of the SNVs we report were predicted to be PTV, which would result in loss of protein function. *In silico* modelling indicates that the 5 missense variants we report would be predicted to interfere with the binding of *MYT1L* to DNA. Both p.His560Tyr and p.Arg569Gln affect conserved amino acids that directly bind to DNA. These variants are likely to disrupt DNA binding. The p.Leu520Pro variant lies at a protein loop which is crucial for the correct folding of the second zinc finger domain; this missense variant is likely to disrupt protein structure and hence DNA binding. His524 and Gly527 are zinc ligands, and any change will disrupt protein structure. The fact that the phenotypes of 2p25.3 deletions and *MYT1L* SNVs overlap supports haploinsufficient loss of function of *MYT1L* as the disease mechanism. By GEP in an *MYT1L* HEK cell line with homozygous *MYT1L* frameshift variants, we demonstrate altered expression of multiple genes implicated in regulation of gene expression and transcription. Haploinsufficiency for *MYT1L* has clear potential to disrupt expression of critical genes during brain development and hence cause a neurodevelopmental disorder.

The expression pattern of *MYT1L* in the human brain reflects the clinical features of individuals with 2p25.3 deletions and *MYT1L* SNVs. The widespread expression of *MYT1L* in brain structures relevant to cognition supports a role for loss of function in the etiology of ID. This is in keeping with the fact that the overwhelming majority of ID and autism genes have widespread expression in the cerebral cortex [33]. The expression pattern of *MYT1L* in human brain also supports a role for the gene in appetite/obesity. *MYT1L* was expressed in multiple hypothalamic nuclei at 15–16 pcw, with significant reduction in expression at 21 pcw. This leads us to hypothesise that *MYT1L* may play a role in the development of the hypothalamus, and that *MYT1L* loss of function may be associated with obesity by impairing development of hypothalamic nuclei. Similar mechanisms operate for other obesity genes such as *SIM1* [8, 9].

To investigate a role for *myt1la/b* in development of the neuroendocrine hypothalamus we generated a knockdown model in zebrafish. Injection of MO against *myt1la* or *myt1lb*, alone or in combination, resulted in a severe loss of expression of *OXT* in the neuroendocrine preoptic area. Knockdown of *myt1la/b* resulted in loss of *AVP* expression in the neuroendocrine preoptic area but not the ventral hypothalamus. This suggests that *myt1la/b* may influence the development of the neuroendocrine preoptic area, but not other regions of the hypothalamus. The neuroendocrine preoptic area is the functional equivalent of the paraventricular nucleus in humans [34]; lesions of which cause hyperphagic obesity [7, 8]. Thus, *MYT1L* CNVs and SNVs may lead to hyperphagic obesity by impairing hypothalamic development.

*SIM1*, functioning with its dimerization partner *ARNT2*, regulates the development of the paraventricular nucleus [7, 8]. *SIM1* deletion heterozygous mice have hypocellularity of the paraventricular nucleus and hyperphagic obesity [7, 8]. To examine an interaction of *SIM1* with *MYT1L* we performed WISH for myt1la in the homozygous *arnt2*^*hi2639T*g^null zebrafish, which has no functional *arnt2* and hence disruption of *sim1a/b* function. The absence of *myt1la* expression in the neuroendocrine preoptic area demonstrates that *MYT1L* lies downstream of *SIM1-ARNT2* in hypothalamic development. Our experiments also indicate that OXT is downstream of *MYT1L*. This suggests that loss of *OXT* may be a final common pathway in genetic forms of obesity, and represent a treatment target in multiple disorders.

In summary, we have identified a series of individuals with *MYT1L de novo* SNVs who present with a syndrome of ID and obesity. Genes involved in nucleosome remodeling, especially those of the neuron-specific Brg1/hBrm Associated Factor (nBAF) complex, have emerged as being central to the pathogenesis of ID [35]. However, *MYT1L* is not known to play a role in nucleosome remodeling, and GEP did not demonstrate that genetic pathways involved in nucleosome remodeling are dysregulated in *MYT1L* knockdown cells. This suggests that the ID observed in patients with *MYT1L* SNVs and CNVs is not related to altered nucleosome remodeling. The mechanism by which *MYT1L* loss of function results in ID is unclear. Murine studies of *MYT1* demonstrate that it promotes neuronal differentiation of neuronal progenitor cells by inhibiting *Notch* signaling [18]. It seems reasonable to suggest that *MYT1L* may perform a similar function in the developing brain and that loss of *MYT1L* function will disrupt this process. The obesity phenotype with *MYT1L* loss of function is associated with disrupted development of the neuroendocrine hypothalamus in zebrafish, manifested by loss of *OXT*. This is similar to the effects of *SIM1* [8, 9] and *POU3F2* loss of function [24], both of which are associated with hyperphagic obesity. *OXT* is emerging as a key neurochemical in both autism and obesity pathogenesis. Polymorphisms in *OXT* and its receptor are associated with autism risk, and intranasal *OXT* improves autism symptoms and imaging abnormalities [36, 37]. *OXT* treatment reduces food intake in humans and in *sim1* mutant mice [38, 39]. In conclusion, we identify *MYT1L*mutations as a cause of syndromic obesity, and establish *MYT1L* as a member of the leptin-melanocortin-SIM1 pathway, with downstream loss of *OXT* associated with *MYT1L* mutations a potential therapeutic target.

## Methods

### Exome sequencing

For probands and their parents in the DDD study, saliva samples were collected (Oragene DNA collection kits, DNA Genotek, Kanata, ON, Canada) and DNA extracted (QIAsymphony, Qiagen, Venlo, Netherlands). Exome sequencing was performed at the Wellcome Trust Sanger Institute with Agilent SureSelect 55MB Exome Plus with Illumina HiSeq to investigate single nucleotide variants (SNVs) and small insertion-deletions (indels) in coding regions of the genome. An automated variant pipleline was used as previously described [4]. Probands were identified with protein altering SNVs in *MYT1L*.

### Genome sequencing

Blood samples were sent for sequencing at the HudsonAlpha Genomic Services Laboratory (http://gsl.hudsonalpha.org). Genomic DNA was isolated from peripheral blood and WGS was conducted to a mean depth of 35X, with >80% of bases covered at 20X. WGS was done on Illumina HiSeq Xs. Reads were aligned and variants called according to standard protocols [40, 41]. A robust relationship inference algorithm (KING) was used to confirm familial relationships [42]. Using filters related to call quality, allele frequency, and impact predictions, we searched for rare, damaging *de novo* variation, or inherited X-linked, recessive, or compound heterozygous variation in affected probands. WGS were carried out under a research protocol and were not completed within a CAP/CLIA laboratory. All variants found to be medically relevant and returnable were validated by Sanger sequencing in an independent CLIA laboratory (Emory Genetics Laboratory) before being returned to participants, although these validated variant results are not CLIA-compliant as the input DNA was originally isolated in a research laboratory. *Ethics approval and consent to participate*: Review boards at Western (20130675) and the University of Alabama at Birmingham (X130201001) approved and monitored this study. *Consent for publication*: A parent or legal guardian was required to give consent to participate in the study and inclusion of their data for publication, and assent was obtained from those children who were capable.

### Ethics statement

The study has UK Research Ethics Committee approval (10/H0305/83, granted by the Cambridge South REC, and GEN/284/12 granted by the Republic of Ireland REC). Written consent taken from all participants and declaration of Helsinki followed.

### *In silico* assessment of *MYT1L* variant pathogenicity

The predicted effect of the *MYT1L* missense variants was examined using SIFT and PolyPhen. Evolutionary conservation of mutated amino acids was assessed by aligning orthologs in Ensembl (http://www.ensembl.org/index.html). The presence of *MYT1L* variants in control populations and *MYT1L* constraint metrics were queried using the ExAC browser (http://exac.broadinstitute.org/gene/ENSG00000186487). SNVs are reported using *MYT1L* Isoform-1 (canonical sequence, 1 186 amino acids) and ensemble transcript ENST00000428368(http://www.ensembl.org/Homo_sapiens/Transcript/Summary?g=ENSG00000186487;r=2:1791242-2331116;t=ENST00000428368).

To investigate the structural effects of the *MYT1L* missense variants, we generated a 3-dimensional model of the 2^nd^ and 3^rd^ zinc finger domains of MYT1L bound to DNA. This was based upon the structure of MYT1 4^th^ and 5^th^ zinc finger domains. The amino acid sequence of MYT1 was first extracted from the report of Gamsjaeger et al [43]. The 4^th^ and 5^th^ zinc finger domains of MYT1 and 2^nd^ and 3^rd^ zinc finger domains of MYT1L aligned well, indicating that the structure of MYT1 is suitable to model the effects of MYT1L missense variants. A 3-dimensional model of the 4^th^ and 5^th^ zinc finger domains of MYT1 bound to DNA was then generated using Pymol (https://www.pymol.org/). The *MYT1L* missense variants were placed at the appropriate residues of this to visualize the structural and hence potential functional, consequences.

### Generation of frameshift mutations in *MYT1L* using CRISPR in HEK cell line

Human embryonic kidney (HEK) cell line HEK-293 (HD PAR-617, ATCC CRL-1573) was confirmed to be triploid at the *MYT1L* locus using SNP 6.0 arrays. *MYT1L* transcript ENST00000399161 was targeted. A guide RNA (gRNA1240) was designed to bind at exon 9 of *MYT1L*, with an adjacent protospacer adjacent motif (PAM) site. HEK-293 cultures were transfected with gRNA1240. Colonies were then genotyped by PCR to identify those with homozygous out of frame variants at the gRNA1240 site. PCR sequencing of genomic DNA demonstrated a 1 base pair insertion of a thymidine base within the gRNA1240 site at allele 1 and 2. PCR sequencing demonstrated a 10 base pair deletion in allele 3. Long range PCR did not reveal any larger deletions across the gRNA site. These sequence variants are predicted to cause a downstream STOP codon at amino acid 115 and 170, respectively.

### Gene expression profiling (GEP)

RNA was extracted using trizol and a standard column based system from the isogenic parental HEK-293 line and the knockout line, each in biological triplicate. Whole genome gene expression profiling was performed using the Clariom S array (affymetrix). Differentially expressed genes were defined as those showing a 2 fold or greater change in expression with a false discovery rate of 2% using the Affymetrix Transcriptome analysis console. Enrichment analysis was performed using Enrichr (http://amp.pharm.mssm.edu/Enrichr/) with a crisp gene set (i.e. no fold change expression assigned to each gene) [24].

### *In silico* assessment of *MYT1L* expression pattern in human brain

The expression pattern of *MYT1L* in adult brain was examined using the Allen brain atlas (microarray data using Agilent 8x60K cDNA chip) [23]. Expression data (Z score normalized) were downloaded for 6 adult brains (donor id 9861, 10021, 12876, 14380, 15496 and 15697) for the frontal cortex, hippocampus, basal ganglia and hypothalamus (at least 3 regions from each anatomical site). Expression levels were compared between each anatomical site using Mann-Whitney U-tests. The expression pattern of *MYT1L* in developing human brain was examined using the brainspan atlas. Microarray data (Z score normalized) was downloaded from 2 donor brains at 15–16 post conception weeks (pcw) (donor id 12840 and 14751) and 2 donor brains at 21 pcw (donor id 12566 and 12690) for the paraventricular nucleus, anterior hypothalamic nucleus, lateral hypothalamic area, dorsomedial hypothalamic area, venteromedial hypothalamic area, posterior hypothalamic nucleus and medial mammillary nucleus. Mann-Whitney U-tests were used to compare median *MYT1L* expression in each hypothalamic nucleus at 15–16 pcw and 21 pcw.

### Generation of zebrafish myt1la/b knockdown

Zebrafish (*Danio rerio*) were raised, maintained and crossed as described [43]]. Development of embryos was at 28°C, and staging was determined by both hours post fertilization (hpf) and morphological characteristics [44]. Embryos were genotyped for the *arnt2*^*hi2639cTg*^ allele as previously described [25]. All procedures were in accordance with NIH guidelines on the care and use of animals and were approved by the Georgetown University Institutional Animal Care and Use Committee, Protocol 11–008.

### Templates, probes and Whole-Mount *In Situ* Hybridization (WISH)

Whole-Mount *In Situ* Hybridization (WISH) using DIG-labeled riboprobes was performed as previously described [44]. Zebrafish *myt1la* and *myt1lb* templates were generated by PCR amplification from 2 dpf zebrafish cDNA and then cloned into pJET1.2 vectors in “backwards” direction. The *myt1a* exon 5 primers were 1aF:CACCACGACAATTATTCTAGTG, and 1aR:CTTTAGGGTAGTAAGCTC. The *myt1b* exon four primers were 1bF: AGAGTGACCATATGAATTGCA, and 1bR: CTGCTGCTGGTTATTGTTGAG. The plasmids were linireized with XbaI and probe was synthesized using T7 RNA polymerase and reagents from the DIG labeling kit (Roche).

### Morpholino oligonucleotide (MO) injections

MO injections for *sim1* were used as previously published [45]. For the *myt1la* and *myt1lb* genes, two MOs for each gene were used to knockdown these proteins. One MO was designed to block translation and the other was designed to block splicing. The following MOs were synthesized by Gene-Tools, LCC: *myt1la* ATG MO, 5’-ACCTCCATCTGAATGCAGTGGTTGA; *myt1la* Splice MO, 5’-GGACAGCTGGAGACAAGAGAAATAA; *myt1lb* ATG MO, 5’-CATCTGCTACATCCACCTCCATCTG; *myt1lb* Splice MO, 5’-ATATTTGTGCCCTCACCTATTTCAT; and *tp53* MO, 5’- GCGCCATTGCTTTGCAAGAATTG. The Standard Control MO from Gene Tools was used as control. Solutions consisting of 4 ng/nl MO plus 0.5% tetramethyl rhodamine dextran in dH20 were microinjected into one to four cell stage embryos.

Images were acquired using a Zeiss Axioplan2 microscope fitted with an AxioCam camera using AxioVision software, or, with a Zeiss stereoscope fitted with a Canon Oneshot digital camera. Digitized images were imported into PhotoShop CS (Adobe Systems Inc, San Jose, CA), contrast and brightness adjusted as necessary. WISH for *myt1la/b* expression was quantified using an ordinal scale: 0 = no staining, 1 = dramatically reduced staining, 2 = normal staining. WISH for oxytocin was quantified as follows: ~30 cells = wild type expression, 5–15 cells = reduced, 1–4 = highly reduced, 0 = no expression.

### Statistical analysis

Statistical differences in green fluorescent protein (GFP) expression in 2 somite embryos was determined using ANOVA, followed by Tukey post-hoc tests for individual groups. Significance of MO induced phenotype categories was evaluated by Ordinal Logistic Regression. The statistical analyses utilized SPSS (version 22) from IBM.

### Internet resources

Decipher (https://decipher.sanger.ac.uk/)

Exome aggregation consortium (http://exac.broadinstitute.org/)

Allen Brain Atlas (http://www.brain-map.org/)

SIFT (http://sift.jcvi.org/)

Provean (http://provean.jcvi.org/index.php)

Ensembl (http://www.ensembl.org/index.html)

Enrichr (http://amp.pharm.mssm.edu/Enrichr/)

Pymol (https://www.pymol.org/)

GTEx portal (https://www.gtexportal.org/home/)

## References

[1] Emerson E, Hatton C, Baines S, Robertson J. The physical health of British Adults with intellectual disability: cross sectional study. Int J Equity Heatlh. 2016; 15: 11. 10.1186/s12939-016-0296-x PMC4719222 26791808

[2] Van der Klaauw AA, Farooqi IS. The hunger genes: pathways to obesity. Cell. 2015;161(1): 119–132. doi: 10.1016/j.cell.2015.03.008 25815990 10.1016/j.cell.2015.03.008

[3] Rosenfeld JA, Patel A. Chromosomal microarrays: understanding genetics of neurodevelopmental disorders and congenital anomalies. J Pediatr Genet. 2017; 6 (1): 42–50. doi: 10.1055/s-0036-1584306 28180026 10.1055/s-0036-1584306 PMC5288005

[4] Wright CF, Fitzgerald TW, Jones WD, Clayton S, McRae JF, Kogelenberg MV, et al Genetic diagnosis of developmental disorders in the DDD study: a scalable analysis of genome-wide research data. Lancet. 2015; 385 (9975): 1305–1314. doi: 10.1016/S0140-6736(14)61705-0 25529582 10.1016/S0140-6736(14)61705-0 PMC4392068

[5] Kaur Y, de Souza RJ, Gibson WT, Meyre D. A systematic review of genetic syndromes with obesity. Obes Rev. 2017; doi: 10.1111/obr.12531 28346723 10.1111/obr.12531

[6] Ramachandrappa S, Raimondo A, Cali AM, Keogh JM, Henning E, Saeed S, et al Rare variants in single-minded 1 (SIM1) are associated with severe obesity. J Clin Invest. 2013; 123 (7): 3042–3050. doi: 10.1172/JCI68016 23778139 10.1172/JCI68016 PMC3696558

[7] Wentzel C, Lynch SA, Stattin EL, Sharkey FH, Anneren G, Thuresson AC. Interstitial deletions at 6q14.1-q15 associated with obesity, developmental delay and a distinct clinical phenotype. Mol Syndromol. 2010; 1 (2): 75–81. doi: 10.1159/000314025 21045960 10.1159/000314025 PMC2941842

[8] Tolson KP, Gemelli T, Gautron L, Elmquist JK, Zinn AR, Kublaoui BM. Postnatal SIM1 deficiency causes hyperphagic obesity and reduced Mc4r and oxytocin expression. J Neurosci 2010; 30(10): 3803–3812. doi: 10.1523/JNEUROSCI.5444-09.2010 20220015 10.1523/JNEUROSCI.5444-09.2010 PMC3285557

[9] Michaud JL, Bouchre F, Melnyk A, Gauthier F, Goshu E, Levy E, et al Sim1 haploinsufficiency causes hyperphagia, obesity and reduction of the paraventricular nucleus of the hypothalamus. Hum Mol Genet 2001; 10 (14): 1465–1473. 11448938 10.1093/hmg/10.14.1465

[10] De Rocker N, Vergult S, Koolen D, Jacobs E, Hoischen A, Zeesman S, et al Refinement of the critical 2p25.3 deletion region: the role of MYT1L in intellectual disability and obesity. Genet Med. 2015; 17 (6): 460–466. doi: 10.1038/gim.2014.124 25232846 10.1038/gim.2014.124

[11] Bonaglia MC, Giorda R, Zanina S. A new patient with a terminal de novo deletion of 1.9 Mb associated with early onset obesity, intellectual disabilities and hyperkinetic disorder. Mol Cytogenet. 2014;7:53 doi: 10.1186/1755-8166-7-53 25126114 10.1186/1755-8166-7-53 PMC4131807

[12] Doco-Fenzy M, Leroy C, Schneider A, Petit F, Delrue MA, Andrieux J, et al Early-onset obesity and paternal 2pter deletion encompassing the *ACP1*, *TMEM18* and *MYT1L* genes. Eur J Hum Genet 2014; 22 (4): 471–479. doi: 10.1038/ejhg.2013.189 24129437 10.1038/ejhg.2013.189 PMC3953915

[13] Rio M, Royer G, Gobin S, de Blois MC, Oziliou C, Bernheim A, et al Monozygotic twins discordant for submicroscopic chromosomal anomalies in 2p25.3 region detected by array CGH. Clin Genet 2013; 84 (1): 31–36. doi: 10.1111/cge.12036 23061379 10.1111/cge.12036

[14] Stevens SJC, van Ravenswaaij-Arts CMA, Jannsen JWH, Wassink-Ruiter JSK, van Essen AJ, Dijkhuizen T, et al *MYT1L* is a candidate gene for intellectual disability in patients with 2p25.3 (2pter) deletions. Am J Med Genet. 2011; 155A(11): 2739–2745. doi: 10.1002/ajmg.a.34274 21990140 10.1002/ajmg.a.34274

[15] Khan K, Rudkin A, Parry DA, Burdon KP, McKibbin M, Logan CV, et al Homozygous mutations in PXDN cause congenital cataract, corneal opacity and developmental glaucoma Am J Human Genet 2011; 89 (3): 464–473. 21907015 10.1016/j.ajhg.2011.08.005 PMC3169830

[16] Romm E, Nielsen JA, Kim JG, Hudson LD. Myt1 family recruits histone deacetylase to regulate neural transcription. J Neurochem. 2005; 93 (6): 1444–1453. doi: 10.1111/j.1471-4159.2005.03131.x 15935060 10.1111/j.1471-4159.2005.03131.x PMC1201409

[17] Vierbuchen T, Ostermeier A, Pang ZP, Kokubu Y, Sudhof TC, Wernig M. Direct conversion of fibroblasts to functional neurons by defined factors. Nature. 2010; 463 (7284): 1035–1041. doi: 10.1038/nature08797 20107439 10.1038/nature08797 PMC2829121

[18] Vasconcelos FF, Sessa A, Laranjeira C, Raposo AASF, Teixeira V, Hagey DW, et al MyT1 counteracts the neural progenitor program to promote vertebrate neurogenesis. Cell Rep. 2016; 17 (2): 469–483. doi: 10.1016/j.celrep.2016.09.024 27705795 10.1016/j.celrep.2016.09.024 PMC5067283

[19] Hempel A, Pagnamenta AT, Blyth M, Mansour S, McConnell V, Kou I, et al Deletions and de novo mutations of *SOX11* are associated with a neurodevelopmental disorder with features of Coffin-Siris syndrome. J Med Genet. 2016; 53 (3): 152–162. doi: 10.1136/jmedgenet-2015-103393 26543203 10.1136/jmedgenet-2015-103393 PMC4789813

[20] Sobreira N, Schiettecatte F, Valle D, Hamosh A. GeneMatcher: a matching tool for connecting investigators with an interest in the same gene. Hum Mut. 2015; 36 (10): 928–930. doi: 10.1002/humu.22844 26220891 10.1002/humu.22844 PMC4833888

[21] Lek M, Karczewski KJ, Minikel EV, Samocha KE, Banks E, Fennell T, et al Analysis of protein-coding genetic variation in 60,706 humans. Nature. 2016; 536 (7616): 285–291. doi: 10.1038/nature19057 27535533 10.1038/nature19057 PMC5018207

[22] GTEx consortium. The Genotype-Tissue Expression (GTEx) project. Nat Genet. 2013; 45(6): 580–585. doi: 10.1038/ng.2653 23715323 10.1038/ng.2653 PMC4010069

[23] Hawrylycz MJ, Lein ES, Guillozet-Bongaarts AL, Shen EH, Ng L, Miller JA, et al An anatomically comprehensive atlas of the adult human brain transcriptome. Nature. 2012; 489 (7416): 391–399. doi: 10.1038/nature11405 22996553 10.1038/nature11405 PMC4243026

[24] Kuleshov MV, Jones MR, Rouillard AD, Fernandez NF, Duan Q, Wang Z, et al Enrichr: a comprehensive gene set enrichment analysis web server 2016 update. Nucleic Acid Res. 2016; 44 (w1): W90–97. doi: 10.1093/nar/gkw377 27141961 10.1093/nar/gkw377 PMC4987924

[25] Kasher PR, Schertz KE, Thomas M, Jackson A, Annunziata S, Ballesta-Martinez MJ, et al Small 6q16.1 deletions encompassing *POU3F2* cause susceptibility to obesity and variable developmental delay with intellectual disability. Am J Hum Genet. 2016; 98(2): 363–372. doi: 10.1016/j.ajhg.2015.12.014 26833329 10.1016/j.ajhg.2015.12.014 PMC4746363

[26] Wang T, Guo H, Xiong B, Stessman HA, Wu H, Coe BP, et al De novo genic mutations among a Chinese autism spectrum disorder cohort. Nat Commun 2016; 7: 13316 doi: 10.1038/ncomms13316 27824329 10.1038/ncomms13316 PMC5105161

[27] Khan MJ, Gerasimidis K, Edwards CA, Shaikh MG. Mechanisms of obesity in Prader-Willi syndrome. Pediatr Obes. 2016 11 10 doi: 10.1111/ijpo.12177 27863129 10.1111/ijpo.12177

[28] Montague CT, Farooqi IS, Whitehead JP, Soos MA, Rau H, Wareham NJ, et al Congenital leptin deficiency is associated with severe early-onset obesity in humans. Nature. 1997; 387(6636): 903–908. doi: 10.1038/43185 9202122 10.1038/43185

[29] Krude H, Biebermann H, Luck W, Horn R, Brabant G, Grüters A. Severe early-onset obesity, adrenal insufficiency and red hair pigmentation caused by POMC mutations in humans. Nat Genet. 1998; 19(2): 155–157. doi: 10.1038/509 9620771 10.1038/509

[30] Farooqi IS, Wangensteen T, Collins S, Kimber W, Matarese G, Keogh JM, et al Clinical and molecular genetic spectrum of congenital deficiency of the leptin receptor. N Engl J Med 2007; 356(3): 237–247. doi: 10.1056/NEJMoa063988 17229951 10.1056/NEJMoa063988 PMC2670197

[31] Elsea SH, Williams SR. Smith-Magenis syndrome: haploinsufficiency of RAI1 results in altered gene regulation in neurological and metabolic pathways. Exper Rev Mol Med. 2011; 13: e14. 10.1017/S1462399411001827 21545756

[32] Voll SL, Boot E, Butcher NJ, Cooper S, Heung T, Chow EW, et al Obesity in adults with 22q11.2 deletion syndrome. Genet Med. 2017; 19(2): 204–208. doi: 10.1038/gim.2016.98 27537705 10.1038/gim.2016.98 PMC5292049

[33] Shohat S, Ben-David E, Shifman S. Varying intolerance of gene pathways to mutational classes explain genetic convergence across neuropsychiatric disorders. Cell Rep. 2017; 18(9): 2217–2227. doi: 10.1016/j.celrep.2017.02.007 28249166 10.1016/j.celrep.2017.02.007

[34] Herget U, Ryu S. Coexpression analysis of nine neuropeptides in the neurosecretory preoptic area of larval zebrafish. Front Neuroanat. 2015; 9: 2 doi: 10.3389/fnana.2015.00002 25729355 10.3389/fnana.2015.00002 PMC4325906

[35] Ronan JL, Wu W, Crabtree GR. From neural development to cognition: unexpected roles for chromatin. Nat Rev Genet. 2013; 14(5):347–359. doi: 10.1038/nrg3413 23568486 10.1038/nrg3413 PMC4010428

[36] Zhang R, Zhang HF, Han JS, Han SP. Genes related to oxytocin and arginine-vasopressin pathways: associations with autism spectrum disorders. Neurosci Bull. 2017; 33(2): 238–246. doi: 10.1007/s12264-017-0120-7 28283809 10.1007/s12264-017-0120-7 PMC5360847

[37] Watanabe T, Abe O, Kuwabara H, Yahata N, Takano Y, Iwashiro N, et al Mitigation of sociocommunicational deficits of autism through oxytocin-induced recovery of medial prefrontal activity: a randomized trial. JAMA Psychiatry. 2014;71(2): 166–175. doi: 10.1001/jamapsychiatry.2013.3181 24352377 10.1001/jamapsychiatry.2013.3181

[38] Maejima Y, Iwasaki Y, Yamahara Y, Kodaira M, Sedbazar U, Yada T. Peripheral oxytocin treatment ameliorates obesity by reducing food intake and visceral fat mass. Aging. 2011; 3(12): 1169–1177. doi: 10.18632/aging.100408 22184277 10.18632/aging.100408 PMC3273897

[39] Kublaoui BM, Gemelli T, Tolson KP, Wang Y, Zinn AR. Oxytocin deficiency mediates hyperphagic obesity of Sim1 haploinsufficient mice. Mol Endocrinol. 2008; 22(7): 1723–1734. doi: 10.1210/me.2008-0067 18451093 10.1210/me.2008-0067 PMC2453606

[40] Li H, Durbin R. Fast and accurate short read alignment with Burrows-Wheeler transform. Bioinformatics. 2009;25(14):1754–60. doi: 10.1093/bioinformatics/btp324 19451168 10.1093/bioinformatics/btp324 PMC2705234

[41] DePristo MA, Banks E, Poplin R, Garimella KV, Maguire JR, Hartl C, et al A framework for variation discovery and genotyping using next-generation DNA sequencing data. Nat Genet. 2011;43(5):491–8. doi: 10.1038/ng.806 21478889 10.1038/ng.806 PMC3083463

[42] Manichaikul A, Mychaleckyj JC, Rich SS, Daly K, Sale M, Chen WM. Robust relationship inference in genome-wide association studies. Bioinformatics. 2010;26(22):2867–73 doi: 10.1093/bioinformatics/btq559 20926424 10.1093/bioinformatics/btq559 PMC3025716

[43] Gamsjaeger R, O’Connell MR, Cubeddu L, Shepher NE, Lowry JA, Kwan AH, et al A structural analysis of DNA binding by myelin transcription factor 1 double zinc fingers. J Biol Chem. 2013; 288(49): 35180–35191. doi: 10.1074/jbc.M113.482075 24097990 10.1074/jbc.M113.482075 PMC3853269

[44] Kimmel CB, Ballard WW, Kimmel SR, Ullmann B, Schilling TF. Stages of embryonic development of the zebrafish. Dev. Dyn. 1995;203(3):253–310. doi: 10.1002/aja.1002030302 8589427 10.1002/aja.1002030302

[45] Westerfield M. University of Oregon Press; Eugene, Oregon: 1995. The Zebrafish Book.

